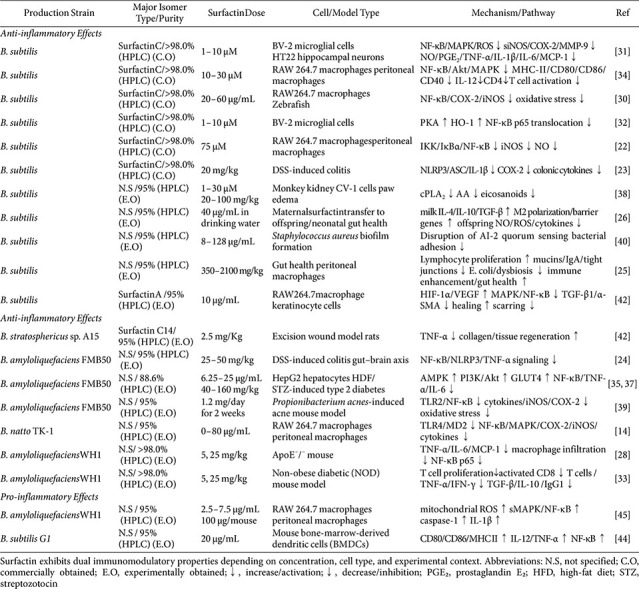# Immunomodulatory Properties of the Cyclic Lipopeptide Surfactin

**DOI:** 10.4014/jmb.2602.02005

**Published:** 2026-04-17

**Authors:** Iskander Madhi, Younghee Kim

**Affiliations:** 1 Department of Integrated Biological Science, Pusan National University, Busan 46241, Republic of Korea; 2 Department of Molecular Biology, College of Natural Sciences, Pusan National University, Busan 46241, Republic of Korea

**Keywords:** Surfactin, Inflammation, Immunomodulation, Therapeutic potential

## Abstract

Surfactin is a cyclic lipopeptide biosurfactant produced by *Bacillus* species that has attracted increasing attention due to its potent immunomodulatory and anti-inflammatory activities. Beyond its well-established surface-active properties, accumulating evidence has demonstrated that surfactin modulates key inflammatory and stress-responsive signaling pathways, including NF-κB, MAPKs, inflammasomes, oxidative stress, and mitochondrial function, across diverse biological systems. These effects translate into protective roles in models of inflammatory, metabolic, microbial, and neuroimmune disorders. However, the biological activity of surfactin is highly context-dependent. Its amphiphilic, membrane-active nature underlies both therapeutic efficacy and dose-limiting toxicity, particularly at higher concentrations or in immune-primed environments. Moreover, variability across studies is strongly influenced by isoform composition, strain-specific biosynthesis, formulation, and potential microbial contaminants, complicating cross-study comparisons and mechanistic interpretation. Toxicological evaluations indicate a favorable safety profile at moderate doses, yet underscore the need for precise dose control and standardized preparations. Recent advances in microbial engineering have enabled isoform-selective biosynthesis and substantially improved production yields. Formulation strategies, including nanoparticle-based delivery, have enhanced stability, reduced cytotoxicity, and expanded therapeutic windows. This review integrates current knowledge of surfactin’s immunological mechanisms, safety considerations, and biotechnological innovations, highlighting critical challenges and opportunities for translation. Collectively, surfactin can be regarded as a tunable molecular platform whose immunological behavior can be modulated through structural control, formulation strategies, and process engineering, thereby supporting its development as a safe and effective therapeutic agent for inflammatory and immune-mediated diseases.

## Introduction

Inflammation is an essential defense program that enables tissues to sense danger, mobilize immune cells, eliminate threats, and initiate repair. This response is coordinated through tightly regulated cytokine and chemokine networks that govern immune activation, cell trafficking, and resolution, ultimately restoring homeostasis when regulation is intact [1]. Central to this balance are interconnected signaling pathways—including nuclear factor kappa B (NF-κB), mitogen-activated protein kinase (MAPK), janus kinase (JAK)-signal transducer and activator of transcription (STAT), and nuclear factor erythroid 2-related factor 2 (Nrf2)/heme oxygenase-1 (HO-1)—alongside regulatory immune cells and metabolic reprogramming mechanisms that ensure inflammatory responses are potent yet self-limiting [2]. When these control systems fail, inflammation becomes chronic, driving tissue damage and disease progression in disorders ranging from autoimmunity and metabolic disease to neurodegeneration and cancer [3]. Accordingly, modern therapeutic strategies aim not merely to suppress inflammation, but to recalibrate immune homeostasis by selectively targeting inflammatory signaling and resolution pathways [4].

Surfactin, a cyclic lipopeptide biosurfactant produced by *Bacillus subtilis* and first isolated in 1968, offers a conceptually distinct approach within this landscape. Its amphiphilic architecture, comprising a fatty acid tail linked to a cyclic peptide head, endows surfactin with exceptional surface activity, self-assembly, ion-chelating capacity, and membrane-interactive properties [5-9]. These physicochemical features underpin its broad utility across environmental remediation, food and agriculture, cosmetics, nanotechnology, and drug delivery systems [5-10], while also driving a diverse biological profile that includes antimicrobial [11], antifungal [12], antiviral [13], anti-inflammatory [14], antitumor [15], and hemolytic activities [16].

This review considers surfactin primarily as an anti-inflammatory modulator, while recognizing that under certain experimental conditions it may also be associated with immune-stimulating responses that require further clarification. By integrating evidences on structural features, isoform diversity, and tissue-specific responses, we provide a balanced interpretation of the varied findings reported in the literature. This integrated view underscores the importance of standardized experimental designs and physiologically relevant models to better define surfactin’s immunological profile and to support its safe and effective application.

### Structural of Surfactin

**Fig. 1:**
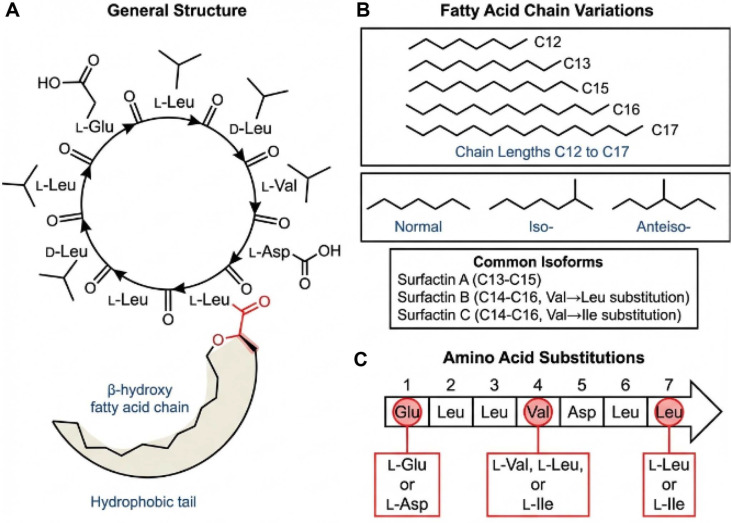
Chemical structures of surfactin isoforms illustrating the conserved heptapeptide core and fatty acid chain variations. (**A**) The general structure of surfactin, a cyclic lipopeptide consisting of a heptapeptide sequence forming a lactone ring with a β-hydroxy fatty acid chain. (**B**) Structural variations in the fatty acid chain length, ranging from C12 to C17, with iso-, anteiso-, and normal branching patterns. (**C**) Amino acid substitutions in the heptapeptide core that generate isoform diversity.

Surfactin exemplifies how defined molecular architecture underpins broad biological functionality. It is a cyclic lipopeptide consisting of a conserved heptapeptide core, most commonly _L_-Glu–_L_-Leu–_D_-Leu–_L_-Val–_L_-Asp–_D_-Leu–_L_-Leu, cyclized through a lactone linkage between the C-terminal leucine and the β-hydroxyl group of a fatty acid chain. As illustrated in Fig. 1, this β-hydroxy fatty acid typically ranges from C13 to C15 in length, although congeners spanning C12-C17 have also been reported [17]. The fatty acid moiety mediates strong hydrophobic interactions with lipid phases, whereas the peptide ring provides spatially organized charged and hydrogen-bonding interfaces. This amphiphilic arrangement promotes a characteristic horseshoe-like conformation, in which hydrophobic side chains form an inward-oriented nonpolar domain, while acidic residues (glutamate and aspartate) remain solvent-exposed, enabling coordination with divalent cations such as Ca^2+^ and Mg^2+^ [17]. Together, these structural features confer a pronounced ability to partition into lipid bilayers, self-associate into micellar or vesicular assemblies, and modulate membrane curvature and tension with high potency at nanomolar concentrations.

A major conceptual advance in the study of surfactin has been the realization that it does not occur as a single molecular entity but rather as an ensemble of closely related isoforms. These congeners, often designated surfactin A through D, with additional minor forms such as surfactin E and F, share a conserved cyclic heptapeptide core, but differ in the length (approximately C12-C17), branching pattern (iso and anteiso), and fine structure of the β-hydroxy fatty acid chain [17]. In most *Bacillus* strains, six to eight dominant isoforms can be detected, with molecular masses typically in the range of ~994–1,036 Da, reflecting combinations of C13-C16 β-hydroxy fatty acids and, in some cases, subtle peptide variants [18, 19]. Surfactin A and B usually carry C13-C14 iso-fatty acids, whereas surfactin C and D incorporate longer and more hydrophobic C15-C16 chains, including anteiso or straight-chain forms [20]. In addition to the dominant C12-C16 isoforms, low-abundance surfactin congeners, including minor longer-chain homologs and sequence variants resulting from single amino acid substitutions (*e.g*., [Val^7^] surfactin), have been reported, further expanding the molecular diversity of the surfactin family [20, 21].

### Anti-Inflammatory Actions of Surfactin in Cellular and Tissue Models

Surfactin exhibits multi-layered anti-inflammatory activity that has been increasingly documented in diverse biological contexts, including the gastrointestinal tract, skin, metabolic tissues, and wound sites (Table 1). Its cyclic lipopeptide architecture enables modulation of membrane microdomains, pattern-recognition receptors, and key signaling pathways, including NF-κB, MAPKs, and cytosolic phospholipase A_2_ (PLA_2_), resulting in the suppression of pro-inflammatory mediators and the promotion of resolution mechanisms [14, 22]. The following discussion synthesizes the current evidences on surfactin’s anti-inflammatory roles in distinct systems, illustrating both shared mechanisms and context-specific outcomes.

**Immunomodulation and gut health.** Surfactin, exerts multifaceted immunomodulatory effects that support gut homeostasis. In murine models of dextran sodium sulfate (DSS)-induced colitis, oral surfactin attenuated intestinal inflammation, preserved barrier integrity and blood–brain barrier function, suppressed tumor necrosis factor-alpha (TNF-α), NF-κB, and nucleotide-binding domain, leucine-rich repeat-containing, pyrin domain-containing 3 (NLRP3) signaling, and beneficially shifted gut microbiota composition, reducing *Proteobacteria* and enriching *Lactobacillus* [23, 24]. Complementary studies have demonstrated that surfactin enhances mucosal immunity by increasing macrophage phagocytosis, lymphocyte proliferation, villus height, and tight junction protein expression (claudin-1, occludin, ZO-1), while elevating secretory IgA and mucins and restoring microbial balance in immunosuppressed mice [25]. In neonatal gastrointestinal development, maternal surfactin supplementation enhanced anti-inflammatory cytokines in breast milk (*e.g*., IL-4, IL-10, transforming growth factor-beta (TGF-β)) and promoted macrophage polarization from pro-inflammatory (M1) to anti-inflammatory (M2), conferring reduced intestinal inflammation and oxidative stress in offspring [26]. *In vitro* fermentation and ecological studies further have revealed that surfactin selectively reshapes gut microbial communities by enhancing beneficial taxa such as *Megamonas*, *Alistipes*, *Methanobrevibacter smithii*, *Lactobacillus*, and *Bifidobacterium*, while suppressing potential pathogens, indicating its capacity to stabilize microbial ecology [27]. In ApoE^-/-^ mice, oral administration of *Bacillus*-derived surfactin reduced atherosclerotic lesion area and macrophage accumulation without affecting serum lipid levels. Surfactin suppressed local expression of pro-inflammatory cytokines (TNF-α, IL-6, IL-1β) and inhibited NF-κB signaling in vascular tissue. Systemically, treatment increased IL-10 levels and promoted anti-inflammatory macrophage polarization, indicating the attenuation of chronic vascular inflammation through immune modulation rather than lipid metabolism [28], suggesting that its effects extend beyond local intestinal immunity to broader gut–associated immune modulation.

**Inflammatory bowel disease (IBD).** Surfactin exhibits pronounced protective effects in experimental models of inflammatory bowel disease, acting through coordinated immunomodulatory, barrier-protective, and microbiota-regulatory mechanisms. In DSS-induced colitis, oral administration of surfactin or surfactin-containing *B. licheniformis* fermented products reduced disease activity, colon shortening, splenomegaly, and histological inflammation, while suppressing NLRP3 inflammasome activation and pro-inflammatory cytokines including TNF-α and NF-κB, demonstrating direct mitigation of intestinal inflammatory pathways [23]. Additional evidence indicates that surfactin can modulate gut microbiota composition, enhance intestinal barrier integrity, and promote anti-inflammatory cytokine expression through maternal milk or *in vitro* fermentation models, emphasizing its capacity to restore microbial homeostasis—a critical factor in IBD pathogenesis [25, 26]. In lipopolysacharride (LPS)-challenged poultry intestine, *Bacillus* fermentates rich in surfactin significantly downregulated TNF-α, IL-1β, and IL-6 expression, supporting localized Toll-like receptor (TLR) antagonism at the mucosal interface [29]. Conceptually analogous hepatic protection observed in copper sulfate-exposed zebrafish revealed that surfactin reduces neutrophil infiltration and suppresses NF-κB p65, cyclooxygenase-2 (COX-2), inducible nitric oxide synthase (iNOS), and cytokines (IL-1β, IL-8, TNF-α), while concomitantly increasing IL-10 levels, which mirror the therapeutic objectives in ulcerative colitis [30]. These multifaceted actions position surfactin as a candidate for barrier-fortifying IBD interventions, warranting dedicated validation in DSS-induced colitis model.

**Neuroinflammation and behavioral disorders.** Emerging evidence indicates that surfactinexerts potent neuroprotective and anti-neuroinflammatory effects through both direct modulation of microglial signaling and indirect gut–brain axis mechanisms. *In vivo*, oral administration of surfactin alleviated DSS-induced colitis in mice while concurrently improving depression-like behaviors, an effect associated with the restoration of gut barrier integrity, modulation of neurotransmitters, and suppression of inflammatory mediators in the brain, including TNF-α, NF-κB, and NLRP3 [24]. Complementing these systemic effects, *in vitro* studies have demonstrated that surfactin attenuates amyloid-β-induced microglial activation, reducing the production of pro-inflammatory cytokines and reactive oxygen species (ROS), thereby protecting neurons from indirect inflammation-mediated toxicity [31]. Similarly, Surfactin inhibited lipoteichoic acid (LTA)-induced pro-inflammatory responses in BV-2 microglia by suppressing NF-κB and STAT1 activation and concurrently upregulating HO-1/Nrf2 signaling, revealing a multi-pathway mechanism that mediates its anti-neuroinflammatory effects [32].

**Chronic inflammation and autoimmunity.** Surfactin exerts potent immunomodulatory effects that are relevant to chronic inflammation and autoimmune processes. In ApoE^-/-^ mice, oral administration of surfactin attenuated chronic vascular inflammation, elevated intestinal IgA level, and increased anti-inflammatory cytokines TGF-β and IL-10, while expanding systemic CD4^+^CD25^+^FoxP3^+^ regulatory T cells and suppressing pro-inflammatory TNF-α and interferon-gamma (IFN-γ), highlighting its capacity to modulate both local and systemic immune responses in chronic inflammatory contexts [28]. Mechanistic studies in LPS-stimulated macrophages have further revealed that surfactin downregulates IFN-γ, IL-6, iNOS, and NO production via NF-κB inhibition, providing cellular evidence for its anti-inflammatory potential [14]. Complementary observations from autoimmune models, including non-obese diabetic mice, indicate that oral surfactin promotes regulatory T-cell expansion and shifts the Th1/Th2 balance, thereby mitigating autoimmune pathology and preserving tissue homeostasis [33]. These effects intersect with the fundamental mechanisms of chronic inflammation, in which regulatory T cells maintain immune tolerance and limit tissue damage, as exemplified in autoimmune diseases and chronic atherosclerosis. At the cellular level, surfactin impairs macrophage pro-inflammatory functions. Treatment of macrophages with surfactin inhibited LPS-induced expression of antigen-presenting molecules, suppressed IL-12 production, and reduced the activation of NF-κB and MAPKs (p38, JNK) and Akt signaling pathways, suggesting broad immunosuppressive effects relevant to autoimmune contexts [34].

**Type 2 diabetes and metabolic inflammation.** Recent studies have highlighted surfactin as a promising modulator of metabolic inflammation and type 2 diabetes. Oral administration of surfactin improved glucose homeostasis in high-fat diet and streptozotocin-induced diabetic mice by reducing pancreatic and intestinal inflammation, oxidative stress, and endoplasmic reticulum stress, while enhancing insulin secretion and intestinal barrier integrity through the activation of phosphoinositide 3-kinase (PI3K)/Akt signaling [35]. Complementary *in vitro* and *in vivo* evidence demonstrated that surfactin ameliorates insulin resistance in HepG2 cells and diabetic mice by increasing glucose transporter 4 expression, suppressing gluconeogenic enzymes such as phosphoenolpyruvate carboxykinase and glucose-6-phosphatase, and decreasing ROS and pro-inflammatory cytokines, resulting in improved fasting glucose levels and reduced weight gain [36]. Emerging studies further indicate that surfactin exerts these metabolic benefits by concurrently activating AMP-activated protein kinase (AMPK) and PI3K/Akt pathways while attenuating MAPK/NF-κB–mediated inflammatory signaling, thereby enhancing glucose uptake in peripheral tissues and dampening chronic metabolic inflammation [37].

**Acne and skin inflammation.** Surfactin's synergistic antimicrobial and anti-inflammatory properties make it particularly suitable for treating inflammatory dermatoses such as acne vulgaris. Topical application in the classic TPA-induced mouse ear edema model demonstrated robust suppression of acute cutaneous inflammation via selective inhibition of cytosolic PLA_2_, blocking arachidonic acid release and consequent eicosanoid-driven edema formation [38]. Topical application of surfactin oleogel alleviates *Propionibacterium acnes*-induced inflammatory acne in mice. Treatment reduced epidermal swelling, oxidative stress, and the expression of pro-inflammatory mediators including TNF-α, IL-1β, iNOS, and COX-2, while inhibiting TLR2-mediated NF-κB activation, underscoring surfactin’s antibacterial and anti-inflammatory efficacy in skin inflammation [39]. Critically, surfactin exhibits potent anti-biofilm and bactericidal activities against acne-relevant pathogens including *Cutibacterium acnes* and *Staphylococcus epidermidis*, enabling the dual-targeting of microbial triggers and host inflammatory responses within pilosebaceous units [40].

**Wound healing and scar reduction.** Surfactin has emerged as a potent promoter of wound healing and modulator of scar formation [41]. Experimental studies have demonstrated that surfactin A accelerates wound closure, enhances angiogenesis through hypoxia-inducible factor 1-alpha (HIF-1α) and vascular endothelial growth factor (VEGF) upregulation, and stimulates keratinocyte migration via MAPK and NF-κB signaling pathways, while also modulating macrophage phenotypes to favor tissue repair [42]. Additional *in vivo* evidence indicates that surfactin’s antioxidant and antimicrobial properties contribute to accelerated epidermal regeneration and improved dermal architecture, highlighting its multifunctional role in tissue repair [43]. Mechanistically, surfactin orchestrates a coordinated healing response by suppressing pro-inflammatory cytokines, promoting macrophage M1-to-M2 transition, and inhibiting fibrosis through the downregulation of alpha-smooth muscle actin and TGF-β1, thereby reducing scar formation.

### Surfactin as a Modulator of Immune Responses

Although surfactin has been predominantly investigated for its anti-inflammatory properties, two studies indicate that, under specific conditions, it can also trigger immune activation, cellular stress, and pro-inflammatory signaling as shown in Table 1. First, *in vitro* study demonstrated that surfactin at 20 μg/mL promotes the maturation of murine dendritic cells, as evidenced by both morphological changes and upregulation of surface maturation markers such as MHC class II and CD40. This phenotypic maturation is accompanied by enhanced functional activity, including increased secretion of pro-inflammatory cytokines IL-6 and TNF-α, augmented chemotactic migration toward CCL19, and a greater capacity to stimulate allogeneic T-cell proliferation [44].

Another report [45] showed that surfactin penetrates macrophages and triggers mitochondrial ROS production, which activates p38 MAPK, JNK, and NF-κB signaling, resulting in TNF-α secretion and the expression of multiple inflammasomes, including NLRP1, NLRP3, IPAF, and AIM2, ultimately promoting IL-1β maturation *in vitro* but inducing only NLRP1 expression *in vivo*. Mechanistically, surfactin behaves as a non-pathogen-associated molecular pattern, enhancing innate immune responses through ROS-dependent pathways that converge on MAPKs and NF-κB, providing a molecular basis for its cytokine-inducing and adjuvant-like activities. The amphiphilic architecture of surfactin is thought to enable its integration into cellular membranes and modulate receptor-associated signaling, including pathways downstream of TLRs, without necessitating direct ligand–receptor binding.

Surfactin is biosynthesized as a heterogeneous mixture of closely related isoforms that primarily differ in the β-hydroxy fatty acid chain length (typically C13-C16), with additional variation arising from the peptide sequence and stereochemistry [17]. These structural differences modulate amphipathicity and lipid interaction properties and have been to influence membrane association and perturbation behavior [46]. Nevertheless, most studies available in the literature do not specify the isoform composition of the surfactin preparations used, thereby limiting direct comparison across reports and possibly contributing to variability in observed immunological effects.

Similarly, the composition of surfactin is further influenced by the producing *Bacillus* strain, as genetic variation within the non-ribosomal peptide synthetase machinery results in strain-specific surfactin profiles [18]. Consequently, surfactin isolated from different microbial sources cannot be assumed to be chemically or functionally equivalent, which is a factor that may substantially affect biological outcomes, particularly in immune-responsive cellular models.

**Fig. 2:**
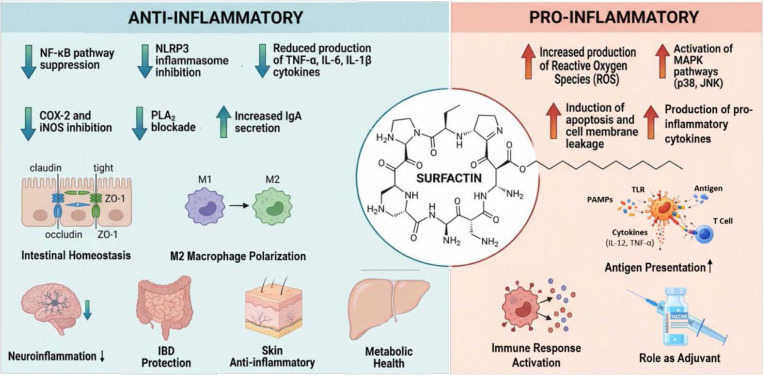
Dual immunomodulatory effects of surfactin. Surfactin exhibits both anti-inflammatory and pro-inflammatory activities. Its anti-inflammatory effects include suppression of the NF-κB signaling pathway and inhibition of the NLRP3 inflammasome, resulting in reduced production of pro-inflammatory cytokines such as TNF-α, IL-6, and IL-1β. These effects are accompanied by downregulation of COX-2 and iNOS, inhibition of PLA2, and enhanced IgA secretion. Collectively, these mechanisms contribute to beneficial outcomes, including maintenance of intestinal homeostasis, M2 macrophage polarization, and attenuation of neuroinflammation, inflammatory bowel disease, skin inflammation, and metabolic dysfunction. In contrast, surfactin can also promote pro-inflammatory responses in a context-dependent manner through increased ROS production, activation of MAPK signaling, induction of apoptosis, and stimulation of proinflammatory cytokine expression. These effects may enhance immune activation, including antigen presentation and adjuvant activity.

In addition, pro-inflammatory responses that might be attributed to surfactin may in some cases reflect contamination with microbial pathogen-associated molecular patterns, such as LTA, introduced during extraction or purification of surfactin. This issue is particularly relevant in macrophage and microglial systems, as these cells are highly sensitive to trace levels of endotoxin or lipoteichoic acid. Therefore, inflammatory effects should be interpreted cautiously unless rigorous purification procedures and appropriate endotoxin controls are clearly documented. Taken together, these considerations indicate that the immunomodulatory properties of surfactin are not attributable to a single event but rather to its complex interference with several key intracellular signaling networks, as summarized in Fig. 2.

### Navigating Surfactin's Therapeutic Potential and Biotechnological Challenges

While surfactin was originally studied for its exceptional surface-active properties, accumulating evidence indicates that it functions as a multifunctional bioactive molecule with context-dependent immunomodulatory and anticancer effects. However, realizing surfactin’s clinical and biotechnological potential necessitates a careful balance between biological efficacy, safety, and scalable production. This section integrates current insights into surfactin’s therapeutic activities, safety profile, and the biotechnological hurdles that must be overcome to translate laboratory findings into real-world applications.

**Safety and toxicology.** The therapeutic index of surfactin reflects dose- and isoform-dependent membrane interactions. Surfactin exhibits a favorable safety profile at moderate doses, but its membrane-active properties necessitate careful toxicological evaluation. Subacute oral administration of surfactin C in rats revealed no overt toxicity up to 500 mg/kg/day, with survival, clinical signs, and hematological parameters unaffected, establishing this level as a no-observed-adverse-effect level. In contrast, higher doses induced dose-dependent hepatocyte necrosis and increased liver weight [47]. Complementary assessments of genetic and developmental toxicity confirmed that surfactin C produced no genotoxic, teratogenic, maternal, or fetotoxic effects in bacterial mutagenesis and rodent developmental assays at doses up to 500 mg/kg/day [48]. Broader reviews of surfactin toxicology emphasize its membrane-disruptive mechanism, which can cause cytotoxicity, hemolysis, and immunogenicity at high concentrations. Rodent studies have reported liver enzyme elevations and gastrointestinal effects above approximately 100 mg/kg [49]. These observations underscore the importance of dose control and further long-term exposure studies.

**Surfactin in therapy: bridging molecular mechanisms and microbial engineering toward clinical applications.** Despite its strong anti-inflammatory potential, the biomedical application of surfactin is constrained by membrane-associated toxicity at higher doses, batch-to-batch isoform heterogeneity, and limited systemic stability due to hydrolysis of the macrolactone ring [50, 51]. These challenges underscore the need for formulation and engineering approaches that control isoform composition, improve solubility, and stabilize the lactone structure.

Isoform standardization is essential for the pharmaceutical development of surfactin, as variations in the fatty acid chain length (C13-C15) and branching significantly alter its physicochemical and biological properties [46]. To ensure therapeutic consistency, clinical programs utilize either purified single-isoform substances or rigorously defined homolog mixtures with strict acceptance criteria. Achieving this precision necessitates high-resolution HPLC or LC-MS analysis, validated quantitative protocols, and the establishment of certified reference standards to ensure inter-laboratory comparability and regulatory compliance [50]. Recent advances in microbial engineering offer powerful solutions by enabling precise control over both the yield and isoform distribution [52]. Through rational genetic engineering of *Bacillus* strains, researchers can manipulate the entire biosynthetic pipeline by optimizing the non-ribosomal peptide synthetase (NRPS) machinery, strengthening regulatory elements, and rewiring host metabolism to funnel key building blocks (amino acids and branched-chain fatty acids) toward surfactin production [52-55]. For instance, using CRISPR interference (CRISPRi) to repress competing amino acid pathways can boost titers nearly fivefold and selectively increase the proportion of specific isoforms, such as the C14 variant [56]. In a landmark demonstration, multistep strain engineering that restored biosynthetic genes and eliminated metabolic competition achieved remarkable production levels of approximately 12.8 g/L from a near-zero baseline [53]. These modifications do more than just increase output; by altering the fatty acid precursor supply, they directly reshape the isoform distribution [54-56]. Since the fatty acid chain length defines surfactin's amphiphilic structure, this control allows the tailored production of surfactant mixtures with optimized physicochemical properties for target applications, marking a shift from simple production boosting to precision molecular design [52].

Nanoformulation strategies complement these upstream advances by enhancing the therapeutic window of surfactin [57]. Encapsulation into biopolymer-based nanoparticles is a promising approach to potentiate anti-inflammatory and antimicrobial activities while providing controlled delivery. In a periodontal disease model, surfactin loaded into κ-carrageenan oligosaccharide-cellulose nanofiber nanoparticles produced a dose-dependent reduction in inflammation in LPS-stimulated human gingival fibroblasts [57]. The optimal formulation significantly lowered key pro-inflammatory mediators, prostaglandin E_2_ by approximately 78% and IL-6 by 75% compared with LPS-only controls, with efficacy comparable to the antibiotic doxycycline. These nanoparticles also markedly decreased oxidative stress and lipid peroxidation, confirming that nanoformulation can amplify surfactin's bioactivity while mitigating toxicity.

Since surfactin is typically produced by Gram-positive *Bacillus* species, LTA and other cell-wall components may be present as non-endotoxin pyrogens. These molecules are not detected by conventional endotoxin assays but can activate inflammatory pathways through TLR2. The Monocyte Activation Test (MAT) has therefore gained attention as a broader pyrogen detection method, and the United States Pharmacopeia (USP) recognizes it as a compendial alternative for detecting pyrogenic substances beyond endotoxin [58]. Although *Bacillus* producers are Gram-positive, exogenous contamination with LPS from raw materials or processing steps can still occur. Even very low concentrations of LPS can activate TLR4-dependent signaling in sensitive immune cells, potentially confounding biological assays and posing safety risks *in vivo* [59]. Consequently, pharmacopeial endotoxin testing according to USP <85> Bacterial Endotoxins Test is required, typically using Limulus Amebocyte Lysate (LAL) or recombinant Factor C assays [60]. Acceptable endotoxin specifications are generally derived from the pharmacopeial threshold pyrogenic dose (K = 5 EU/kg body weight for parenteral administration and 0.2 EU/kg for intrathecal administration), with the final endotoxin limit determined using the K/M calculation defined in USP <85> [60].

The regulatory translation of surfactin into clinical applications requires adherence to established pharmaceutical quality and safety frameworks. Although *B. subtilis* is widely regarded as a safe production organism and has been granted Generally Recognized as Safe (GRAS) status for certain applications, purified surfactin must undergo independent safety and quality evaluation according to its intended therapeutic indication, route of administration, and dosing regimen. During drug development, quality specifications for active pharmaceutical ingredients (APIs) are progressively refined as manufacturing processes mature and analytical characterization improves. These expectations are defined within international regulatory frameworks, including ICH Q7 Good Manufacturing Practice (GMP) Guide for APIs [61], ICH Q6A Specifications: Test Procedures and Acceptance Criteria for New Drug Substances and New Drug Products [62], and ICH Q3A(R2) Impurities in New Drug Substances / ICH Q3B(R2) Impurities in New Drug Products [63], which collectively require well-controlled impurity profiles, validated analytical methods, and consistent GMP manufacturing. Surfactin intended for pharmaceutical development would therefore be expected to meet comparable quality standards.

## Conclusion

This review positions surfactin as a multifunctional immunomodulatory lipopeptide whose biological activity arises from its distinctive amphiphilic structure and isoform diversity. Across multiple tissues, including the gastrointestinal tract, skin, metabolic organs, vasculature, and central nervous system, surfactin consistently modulates inflammatory signaling networks such as NF-κB, MAPKs, inflammasomes, and oxidative stress pathways. In parallel, it promotes barrier integrity, regulatory immune responses, and tissue homeostasis, underscoring its potential as a broadly acting anti-inflammatory agent rather than a single-pathway inhibitor.

However, surfactin’s immunological effects are highly context dependent. Under specific experimental conditions, its membrane-active properties can provoke immune activation through mitochondrial dysfunction, ROS generation, and downstream inflammatory signaling. Variability across studies could be further influenced by isoform composition, strain-specific biosynthesis, formulation, and the presence of microbial contaminants, highlighting the necessity for rigorous purification and experimental standardization. Advances in microbial engineering could allow isoform-selective biosynthesis and scalable production with improved batch consistency, while formulation strategies, particularly nanocarrier-based delivery, could enhance stability, mitigate cytotoxicity, and expand therapeutic windows. Integration of these approaches with regulatory frameworks, including GMP-compliant manufacturing and pharmacopeial quality control, will be essential for clinical translation.

In conclusion, surfactin should be viewed not merely as a bioactive biosurfactant but as a tunable molecular platform whose immunological behavior can be precisely modulated through structural control, formulation strategies and process engineering. Future studies incorporating well-standardized experimental systems will be essential to systematically define surfactin’s context-dependent mechanisms and to establish its potential as a safe and effective therapeutic agent for inflammatory and immune-mediated diseases.

## Figures and Tables

**Table 1:** Immunomodulatory effects of surfactin: a comprehensive summary of experimental models, molecular mechanisms, and key findings